# Supplementary material for: Genetic Signature and Serocompatibility Evidence for Drug Resistant Campylobacter jejuni
Source: Antibiotics (Basel). 2022 Oct 17;11(10):1421. doi: 10.3390/antibiotics11101421 (PMC9598221; doi:10.3390/antibiotics11101421)
Supplement: Supplementary file 1 [file antibiotics-11-01421-s001.zip › antibiotics-1966720-supplementary.pdf]

Supplementary Materials

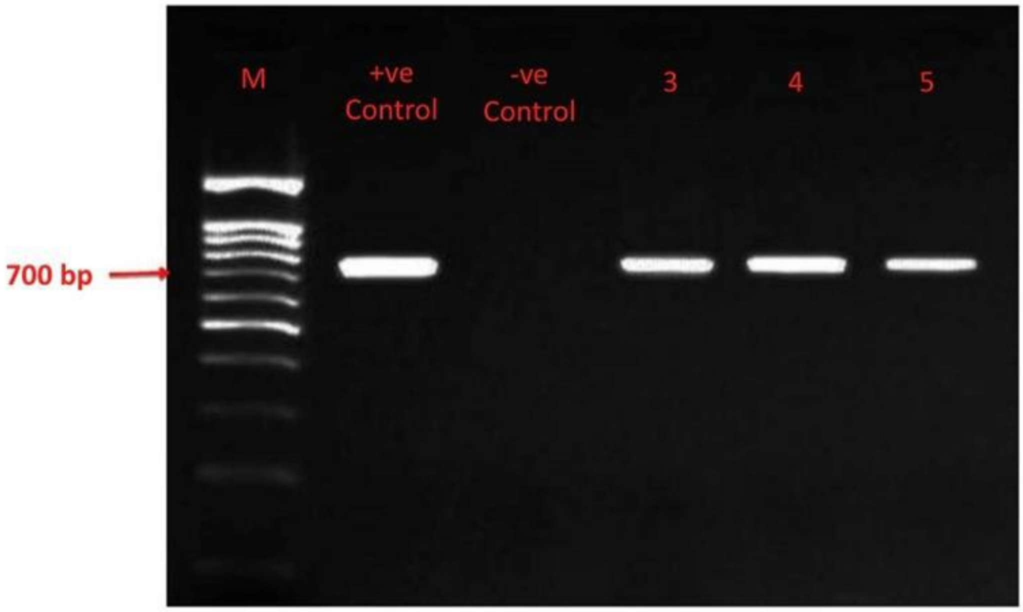

Figure S1. The PCR amplification of *hipO* gene showed 700 bp product. Lane M, 100 bp DNA ladder; lane 1, positive control (DNA from *C. jejuni* ATCC 33291); lane 2, negative control; lanes 3-5 showed the expression of *hipO* in *Campylobacter jejuni* isolates.

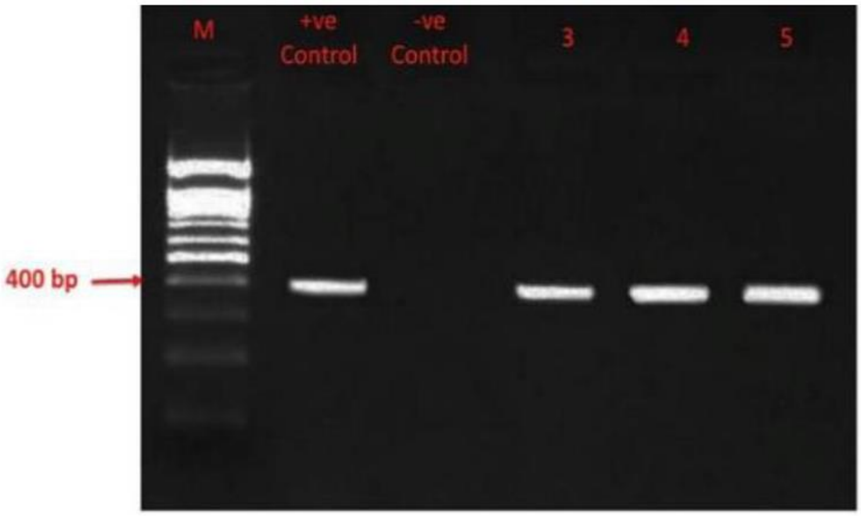

Figure S2. The PCR amplification of *cadF* gene showed 400 bp product. Lane M, 100 bp DNA ladder; lane 1, positive control (DNA from *C. jejuni* ATCC 33291); lane 2, negative control; lanes 3-5 showed the expression of *cadF* in *Campylobacter jejuni* isolates.

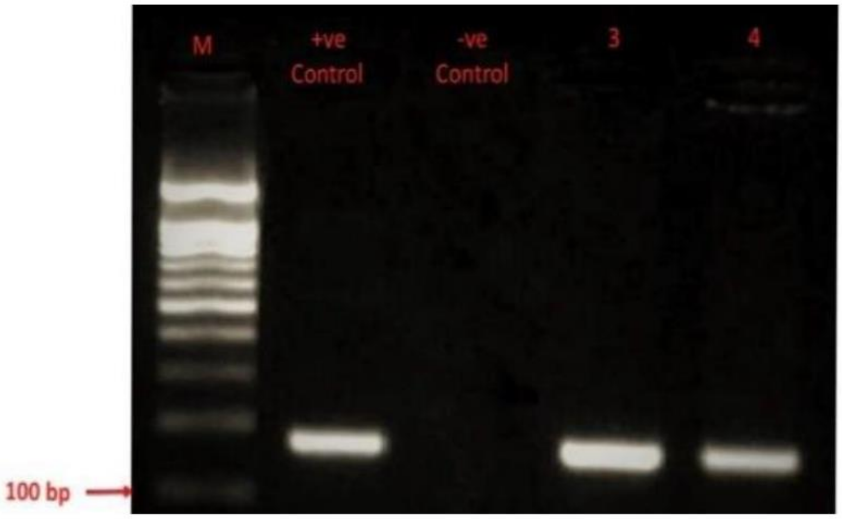

Figure S3. **PCR amplification of *dnaJ* gene showed a 177bp product. Lane M, 100 bp DNA ladder; lane 1, positive control (DNA from *C. jejuni* ATCC 33291); lane 2, negative control; lanes 3 and 4 showed the expression of 177bp in *Campylobacter jejuni* isolates.**

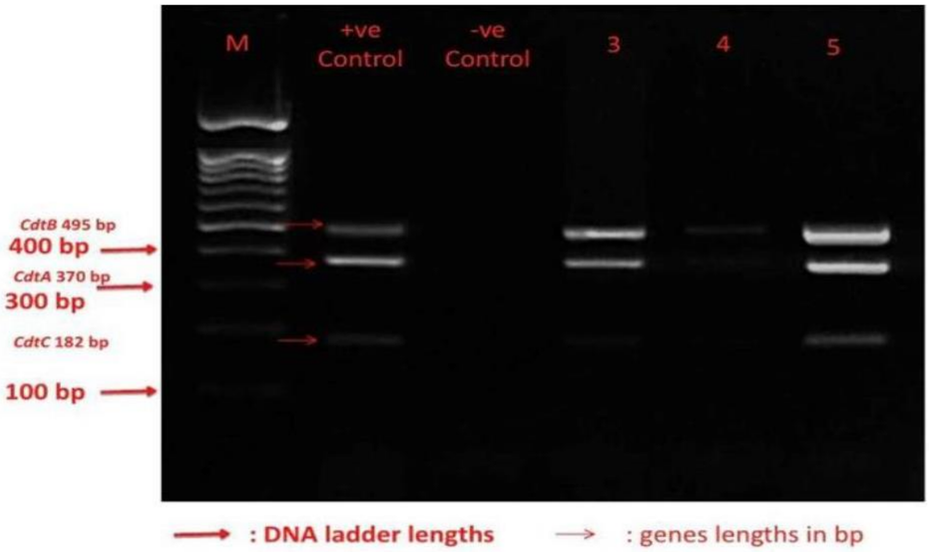

Figure S4. **Multiplex PCR amplification of *cdtA, cdtB, cdtB* produce amplicons having 370 bp, 495bp, and 182 bp, respectively. Lane M, 100 bp DNA Ladder; lane 1, positive control (DNA from *C. jejuni* ATCC 33291); lanes 3-5, positive *Campylobacter jejuni* for *cdt* genes.**
